# Supplementary material for: Are carbon emissions trading and green financial instruments synergistic? -Comprehensive quantitative research based on content analysis
Source: PLoS One. 2024 Mar 7;19(3):e0298601. doi: 10.1371/journal.pone.0298601 (PMC10919601; doi:10.1371/journal.pone.0298601)
Supplement: S1 Data — (ZIP) [file pone.0298601.s001.zip › Data/地级市Z_F工作报告.zip!/╔╜╬≈/╘╦│╟╩╨/└┤╫╘╣1⁄2╓┌║┼í╛┬φ┐╦╩2╛▌═°í┐_2016.docx]

各位代表：

现在，我代表市人民政府向大会报告工作，请予审议，并请市政协委员和其他列席人员提出意见。

一、“十二五”时期国民经济和社会发展回顾

“十二五”时期，是我市发展很不平凡的五年。面对特殊历史时期和持续加大的经济下行压力，市委、市政府团结带领全市人民，认真贯彻党的十八大、十八届三中、四中、五中全会精神，紧紧围绕中央“四个全面”战略布局和省委“六大发展”总体要求，全面落实工业集群化、农业现代化和新型城镇化“三个方案”，大力实施稳增长调结构“三个一百”，攻坚克难，扎实工作，全市经济社会发展取得了新成效。

(一)全力以赴稳增长，经济实力显著增强。面对经济下行压力加大的严峻形势，我们认真落实国家、省稳增长、促改革、调结构、惠民生各项政策措施，千方百计抓投资、上项目、保运行，经济发展迈上新台阶。全市地区生产总值突破千亿元大关，2015年达到1174亿元，总量在全省的排名由“十一五”末的第五名提升为第三名;五年累计达到5637亿元，超过“十五”“十一五”10年总和，年均增长7.8%。财政总收入突破百亿元大关，2015年达到105.5亿元，年均增长5.6%;一般公共预算收入达到56.3亿元，年均增长9.6%。固定资产投资迈上千亿元台阶，2015年达到1370.7亿元，是2010年的2.3倍，年均增长 21.2%。社会消费品零售总额逐年递增，2015年达到661亿元，是2010年的1.9倍，年均增长13%。外贸进出口总额平稳增长，2015年达到 12亿美元，是2010年的1.1倍，年均增长2.7%。城乡居民收入较快增长，2015年城镇居民人均可支配收入达到24049元，年均增长12%;农村居民人均可支配收入达到 8718元，年均增长12.9%。

(二)坚定不移促转型，集群化发展态势良好。按照“全市经济一盘棋，错位发展、差异竞争、优势互动”的发展思路，大力推进园区化发展、集群化招商，成效明显。2015年，全市”5+15″园区规模以上工业企业完成工业总产值955.3亿元，占全市工业的73.5%，比上年提高了3.4个百分点;九大产业集群24个板块完成工业总产值754.5亿元，占全市工业的58%。运城铝工业基地被国家工信部授予“国家新型工业化产业示范基地”。工业园区和产业集群成为拉动全市工业增长的主引擎和重要支撑。大运汽车、华泽铝业、华圣铝业、亚宝药业、万荣汇源等龙头企业引领集群发展，100家重点企业产值占到全市规上工业的70%。新型化工、新能源、新材料、农产品加工等新兴产业发展提速。中小企业发展到1.3万家，2015年实现工业总产值640亿元。360小说

(三)持之以恒抓投资，项目建设成效明显。坚持项目建设“六位一体”工作机制，严格落实项目包联责任制，毫不放松推进项目建设。五年共实施重点项目 1300余项，机场新航站楼、大西客专运城段、侯西铁路电气化复线改造建成运营，蒙西至华中铁路运城段开工建设。运城机场通航29个城市，旅客吞吐量突破百万人次。基础设施建设不断加强。水利上，累计完成投资104.4亿元，是“十一五”时期的3.8倍。电力上，电网总投资49.9亿元，比“十一五”增长 50.8%，电网结构不断完善，供电保障能力显著增强。交通上，闻合、河运、运宝3条高速公路相继建成通车，高速公路总里程超过600公里。完成农村公路建设改造2768公里和3194个建制村2.2万公里街巷硬化。

(四)坚持不懈强基础，城乡一体化协调推进。按照工业集群化、集群园区化、园区社区化、社区城镇化、土地规模化、城乡生态化“六化”衔接，工业化、信息化、城镇化、农业现代化“四化”互动的思路，推动中心城市、大县城、小城镇和新农村统筹协调发展。农业现代化步伐加快。全市粮食总产连续四年稳定在30 亿公斤以上，2015年达到32.1亿公斤，是2010年的1.3倍。肉、蛋、奶产量分别比“十一五”末增长72%、61.5%、8.5%。土地规模流转速度加快，达到212.8万亩，占全市家庭承包土地面积的27%。省级龙头企业发展到70家，国家级龙头企业发展到7家。农产品加工业销售收入完成 251.7亿元，是2010年的2.6倍。建设各类现代农业示范园区443个，完成投资111.6亿元。农民专业合作社达到11475个，是2010年的 2.7倍。水果产量达到600万吨，其中出口13万吨，尤其是运城苹果代表中国首次出口美国，标志着运城苹果具备了通向世界高端市场的质量技术标准。蔬菜产量达460万吨，比“十一五”末增长40%。主要作物耕种收综合机械化水平达70.3%，比2010年提高了17.8个百分点。农田水利末级渠系配套建设增加到15711公里，水地面积达到631万亩，农村人口人均实现两亩水浇地目标。改造中低产田25.5万亩，建设高标准农田43万亩。新型城镇化稳步推进。编制完成了《运城市城市总体规划2011-2030》和15项专项规划。城乡基础设施建设进一步加强，“十二五”期间，全市公用基础设施完成投资 124.4亿元，城市供气、供热、污水处理、供水普及率分别达到90%、77%、91.7%、95%。中心城市荣获全省文明城市、园林城市，学苑北路立交桥、飞雁桥、河东东街延长线等一批城建重点工程建成投入使用。盐临夏一体化和大县城建设步伐加快，20个建制镇入围全国重点镇。全市城镇化率达到 46.1%，比2010年提高8.5个百分点。信息化和智慧城市建设扎实推进。围绕九大产业集群，打造工业化与信息化融合示范标杆，中铝山西分公司成为全国首批示范企业，大运汽车、亚宝药业和中车永济电机成为国家“两化”融合试点企业。“两化”融合贯标体系的建设，带动和促进了全市“两化”融合整体水平的提升。创新智慧城市建设模式，采取政府购买公共服务的方式，由中兴集团投资建设的运营中心、云计算数据中心加快建设，智慧政务、教育、食药、城管、医疗五大基础应用平台建设进展顺利，成为全国首批信息惠民示范城市和全省“互联网+城市服务”智慧城市首批上线城市。

(五)发挥优势求突破，服务业发展势头强劲。全市第三产业增加值比重超过第二产业，占比达到46.1%，比2010年提高了6.8个百分点。文化旅游产业快速增长。“古中国”国际旅游目的地的打造，得到学术界的高度认同和旅游界的强烈反响，激发了开发商的投资热情。关帝庙、盐池三禁门、李家大院、永济水峪口、平陆大天鹅景区、芮城圣天湖景区、绛北大峡谷、垣曲历山等重点文化旅游集聚区建设提档升级，关圣文化建筑群申遗工作积极推进。2015年接待游客是2010年的2.6倍;实现旅游总收入326.9亿元，是2010年的3.2倍。全市各类文化企业达到2100余家，总产值达40多亿元。国家级文化产业基地2家，省级5家。国家级非物质文化遗产达到25项，省级174项，均居全省第一。金融产业成为新的支柱产业。2015年，金融业实现税收6.9亿元，同比增长57.5%，占总税收的7.9%;实现增加值67.3亿元，同比增长16.8%，占全市GDP的5.7%，超国际认定的5%标准，金融业已经成为我市经济的支柱产业。永东化工在深交所中小板上市，长荣农科、寰烁科技、宏安翔科技等企业在新三板成功挂牌。银行业金融机构达到13家，保险机构35家，证券机构6家，晋陕豫金三角金融中心即将建成运营，金融服务体系进一步完善。金融创新力度加大。在全省率先成立“企业应急转贷资金平台”，规模1.3亿元，资金周转额累计13亿元，有效缓解了企业资金周转困难，全省推广了我市做法。创新财政投入模式，成立“运城市产业引导基金”，运城市“河东兴农基金”全面运行。采取有力措施化解金融风险，备受关注的海鑫集团破产重整案取得成功，正在积极组织复产。商贸物流规模扩大。规模连锁企业164家，占到商贸流通企业的30%。盐湖晋善晋美、美特好商超物流中心等项目加快建设。品牌建设成果丰硕。中国驰名商标达到28件，位居全省首位，山西省著名商标达到165件，成为全省唯一的国家商标战略实施示范城市。

(六)持续推进造林绿化，生态环境进一步改善。造林绿化力度加大。“十二五”期间，全市共完成绿化造林217.3万亩，森林覆盖率达到31%。夏县、芮城、临猗、垣曲、平陆、绛县等6个县先后被省政府授予“山西省林业生态县”称号。污染减排工作扎实推进。万元生产总值综合能耗、主要污染物减排等约束性任务全面超额完成。淘汰落后产能企业77家，涉及产能800多万吨。工业固废综合利用率达到70%，高于全省平均水平。国家新能源示范城市、全省循环经济试点市、芮城县国家级生态文明先行示范区、万荣和垣曲低碳试点县建设积极推进。涑水河、姚暹渠“一河一渠”生态环境综合整治和汾河、盐湖、伍姓湖、黄河湿地、城市水源地等水生态保护成效显著。列入“十二五”重点流域水污染防治规划的31个治理项目全部完成，74个工业监管类项目稳定达标排放。

(七)深入推进改革开放，发展活力不断增强。重点领域改革全面推进。政府职能加快转变，全面完成新一轮市县政府机构改革，市政府工作部门精简为32 个。事业单位分类改革稳步推进。共取消、下放和调整行政审批项目141项，权力清单、责任清单、负面清单制度全面推行。国企改革不断深化，通过改制、破产、重组、划转等形式完成改制企业17户。商事制度改革、食品药品、新型医改、公务用车等重点改革扎实推进。对外开放水平不断提升。

招商引资成果丰硕。创新改进招商引资方式，持续开展小分队、专业化、定向化、集群化、产业链精准招商，五年累计引资到位2906亿元。外贸外经企业发展到468家，对外贸易国家和地区达到121个。“十二五”累计新批外资企业20户，实际利用外资2.6亿美元。机场航空口岸、运城海关、运城商检相继建成投入使用或即将投运。区域合作持续扩大。晋陕豫黄河金三角区域合作上升为国家战略，《运城市贯彻落实区域合作规划实施方案》积极推进，三省四市检验检疫、果业协作、文化旅游、公共卫生、基础设施、生态环境等区域合作力度不断加大、领域不断拓展。与天津市南开区、陕西省西安市、河南省济源市签订经济合作战略协议，与黑龙江黑河市、伊春市，浙江省杭州市缔结为友好城市。

(八)竭尽全力惠民生，人民生活水平有效改善。民生支出大幅增长。“十二五”期间，民生支出年均增长17.2%，高于一般预算支出2个百分点。 2015年，一般公共预算支出276.9亿元，其中民生支出233.8亿元，占总支出的84.4%。在财政收入较为困难的情况下，千方百计保证国家、省、市各项民生政策及时兑现，特别是做到了与群众利益直接相关的各种工资福利待遇不拖不欠。各类教育协调发展。平陆、垣曲、永济等7个县(市)高标准通过了国家义务教育发展基本均衡县达标验收，新改扩建公办幼儿园435所，连续两年代表山西参加“中国汉字听写大会”，为运城赢得荣誉。中等职业教育免学费和助学金政策惠及学生10.5万人次，运城学院新校区、运城职业技术学院等新校建设提档升级。医疗卫生事业扎实推进。城镇基本医疗保险参保88万人，全市新农合参保率达到99.3%。市第一医院、市第三人民医院加快建设。社会保障不断加强。城乡养老、城乡低保、农村五保等标准逐年提高，覆盖面持续扩大，机关事业单位养老保险制度改革全面落实。连续5年提高低保标准，城市由每人每月217元提高到413元，农村由99.7元提高到223.9元。农村五保集中供养标准由每人每年2008元提高到4900元，分散供养标准由每人每年1527元提高到3246元。5年累计减贫17.2万人。城乡居民社会养老保险参保 281.6万人。各类保障性住房开工建设84698套，建成60078套。完成农村危房改造5.4万户。农村“五个全覆盖”和农村“五件实事”全面完成。新增就业持续增加。“十二五”期间，城镇新增就业29.7万人，转移农村劳动力44.3万人，失业人员再就业7.4万人，就业困难人员就业2.3万人，创业带动就业6.5万人。城镇登记失业率控制在4%以内。先后建成一批高技能人才培训基地、技能大师工作室，累计开展各类职业技能培训58.5万人次。科技、文化等社会事业全面发展。有效发明专利达到537项。高新技术企业达到63家。培育了中车永济电机和南风化工等7家省级创新型企业。民营科技企业达 77家。文化事业繁荣发展，“戏曲惠民、欢乐百姓”“群文风采大展赛”等系列活动惠及城乡百姓。市群艺馆改扩建成为全省功能最全的市级群众文化乐园。新建了县级图书馆6个、文化馆11个、乡镇文化站136座、社区文化活动中心121个、农家书屋3189个。文化工作受到国家表彰。体育工作成绩斐然，群众性体育蓬勃发展，体育产业再攀新高。档案工作基础扎实，市档案馆升级为省一级馆，新建7个县级馆。食品安全放心城市创建工作成效明显，“人大抓监督、政府抓创建、部门抓监管、行业严自律、社会共参与”的食品安全工作新格局初步形成。安全生产形势稳定好转，连续5年没有发生重大以上安全事故。

(九)加强党风廉政和民主法治建设，社会保持和谐稳定。深入开展党的群众路线教育实践活动、“三严三实”专题教育和学习讨论落实活动，严格执行中央 “八项规定”、坚决反对“四风”。狠抓党风廉政建设和反腐败斗争，大力压减“三公”经费，行政监察和审计监督力度不断加大。自觉接受人大法律监督、政协民主监督和社会舆论监督，认真听取各民主党派、工商联和无党派人士意见，支持工会、共青团、妇联等人民团体发挥作用。深入开展“六五”普法。社会主义核心价值观教育深入推进。精神文明建设不断加强。加强学习型政府建设，定期举办“政府大讲堂”活动，引导广大干部特别是领导干部不断转变观念、开阔视野、提升能力。社会治安防控治理和信访工作全面加强，公共法律服务体系不断完善。认真贯彻党的民族宗教政策，民族团结、宗教和谐局面进一步巩固。双拥工作和军民融合式发展深入推进。统计、人防、地方志、妇女儿童、老龄、残疾人等工作都取得新成绩。应急管理和防震减灾能力不断增强，社会大局和谐稳定。

五年来，我们深切感受到，加快运城经济社会持续健康发展，必须坚持“想透、说清、干实”，解放思想、尊重规律、更新观念，善于把中央、省的决策部署与运城发展的实际紧密结合，创造性地谋划发展、推动工作;必须坚持开放理念，拓展发展空间，以开放倒逼改革，以开放推动转型;必须坚持问题导向，创新工作机制，找准经济社会发展的突出矛盾，特别是把扩大固定资产投资作为推动转型发展的关键;必须坚持一切从实际出发，以只争朝夕的状态，功成不必在我的境界，实事求是的精神和抓铁有痕、踏石留印的作风，多做打基础、利长远、惠民生、可持续的事;必须坚持为民务实清廉，加强政府自身建设，敬畏法纪，勇于担当，不断提升政府的执行力和公信力。

各位代表，“十二五”经济社会发展取得的成绩来之不易。这是省委、省政府和市委坚强领导、科学决策的结果，是市人大、市政协大力支持、有效监督的结果，是全市广大干部群众同心同德、团结拼搏的结果。在此，我代表市人民政府，向全市广大干部群众，向大力支持政府工作的人大代表、政协委员、各民主党派、工商联、无党派人士和人民团体，向驻运部队、武警官兵和公安干警，向所有关心支持运城发展的社会各界人士，表示衷心的感谢和崇高的敬意!

在肯定成绩的同时，我们也清醒地认识到，我市的经济社会发展还存在一些不容忽视的困难和问题，主要表现在：面对经济新常态，我们的思维方式、工作方法和工作能力还很不适应，与认识新常态、适应新常态、引领新常态的要求还有很大差距;如何把“创新、协调、绿色、开放、共享、廉洁和安全”的发展理念与运城的经济社会发展实际更加紧密地结合起来，特别是在推动创新与开放发展上找到突破点，还需要下更大的功夫;经济结构不优，传统产业占比较重，新兴产业发展不足，产业集群规模小，竞争力不强，去产能、去库存、去杠杆、降成本、补短板的任务极为繁重;新型城镇化步伐不快，城镇化率低于全国、全省10个百分点左右;文化旅游产业占比较小，与厚重的“古中国”文化底蕴极不相称;经济发展的质量和效益还不高，财政规模小，人均GDP和城乡居民收入与全国、全省及发达地区相比差距较大;政府职能转变仍显滞后，“四风”问题时有反弹，干部作风转变还不到位，不能为、不想为、不敢为的问题较为突出，党风廉政建设和反腐败斗争任务繁重。对这些问题，我们一定要在今后的工作中精准发力、综合施策、全力突破。二、“十三五”时期经济社会发展的指导思想和目标任务

“十三五”时期是全面建成小康社会的决胜阶段，谋划布局“十三五”发展、做好“十三五”工作意义重大。全市经济社会发展的指导思想是:高举中国特色社会主义伟大旗帜，全面贯彻党的十八大和十八届三中、四中、五中全会精神，以马克思列宁主义、毛泽东思想、邓小平理论、“三个代表”重要思想、科学发展观为指导，深入贯彻习近平总书记系列重要讲话精神，遵循“四个全面”战略布局，按照省委十届七次全会安排部署，切实强化创新、协调、绿色、开放、共享、廉洁和安全发展理念，坚持发展是第一要务，以提高发展质量和效益为中心，以落实晋陕豫黄河金三角区域合作规划为突破口，突出中心城市的引领带动作用，坚持开放带动、拓展发展新空间，坚持改革推动、培育发展新动力，坚持创新驱动、厚植发展新优势，努力把运城建成新型工业大市、现代农业强市、文化旅游名市和晋陕豫黄河金三角区域性中心城市，确保如期全面建成小康社会。

“十三五”时期，我市经济社会发展的主要目标是：创新转型发展促进经济较快增长。充分发挥自身比较优势，加快转型发展，主要经济指标增速高于全国全省平均水平。工业集群化水平显著提升，重点产业集群和产业板块的竞争力和影响力明显增强。农业现代化迈上新台阶，九大农业板块、五大基地和“百园千村”建设取得明显成效。新型城镇化加速推进，具有运城特色的“六化”衔接、“四化”互动新型城镇化路径更加成熟。信息化建设初具规模，智慧政务、智慧医疗、智慧教育等平台服务功能完备，信息化成果广泛惠及城乡居民，工业化、信息化、城镇化、农业现代化融合发展。传统产业优化升级，新兴接替产业规模扩大，文化旅游产业进一步壮大，服务业比重不断提高。科技、消费对经济增长贡献明显加大。人才强市建设迈出新步伐。全面深化改革、全方位扩大开放格局基本形成。晋陕豫黄河金三角区域协调发展试验区推进工作取得重大进展，区域一体化发展格局初步形成。重点领域和关键环节改革取得重大成果。开放型经济和对外合作机制基本形成，对外开放的广度和深度不断拓展。人民生活水平稳步提升。就业比较充分，教育、文化、社保、医疗、住房等公共服务体系更加健全，基本公共服务均等化水平显著提高。教育现代化取得重要进展，劳动年龄人口受教育年限明显提高，收入差距缩小，中等收入人口比重上升，现行标准下的贫困人口全部稳定脱贫，贫困县全部摘帽。生态环境质量显著改善。主体功能区分布和生态安全屏障基本形成，生产方式和生活方式绿色低碳化水平明显提高。能源资源使用效率大幅提高，能耗和水资源消耗、建设用地、碳排放总量得到有效控制，主要污染物减排完成省下达任务。黄河流域综合治理取得实质性进展。大气、水、土壤污染治理取得新成效。森林覆盖率不断提高。民主法治建设深入人心。人民民主不断扩大，法治政府基本建成，司法公信力明显提高，民主法治更加健全，社会治理能力和水平不断提高，社会更加和谐稳定。全体市民素质普遍提高。中国梦和社会主义核心价值观更加深入人心，爱国主义、集体主义、社会主义思想广泛弘扬，向上向善、诚信互助的社会风尚更加浓厚，人民思想道德素质、科学文化素质、健康水平明显提高。公共文化服务体系基本建成，文化旅游产业成为支柱产业。

“十三五”时期，我市经济社会发展的主要任务是：

(一)坚持创新发展，推动经济转型升级。坚持把创新作为引领发展的第一动力和核心。围绕建设新型工业大市，抢抓供给侧改革机遇，加快传统产业优化升级、新兴产业规模扩张、成长性产业培育壮大、优势产业集群发展，实现工业经济转型升级。围绕建设现代农业强市，推进土地规模化、组织企业化、技术现代化、经营市场化，提高农业质量、效益和竞争力。围绕建设文化旅游名市，整合文化旅游资源，大力发展文化旅游产业、现代金融业和商贸物流业，不断提升服务业发展水平。围绕建设黄河金三角区域性中心城市，发挥区位、交通、教育、政策等优势，优化资源配置，完善城市功能，促进人流、物流、信息流、资金流等各种生产要素在运城集聚，激发市场活力和社会创造力。

(二)坚持协调发展，构建均衡发展格局。坚持发展经济与改善民生并重，在加快发展经济的同时，更加注重社会事业发展，不断加大对民生工程和社会事业的投入，促进经济社会协调发展。坚持城乡统筹发展，实现以城带乡、以工促农、工农互惠，积极推进城乡规划、市场培育、基础设施、公共服务、行政管理一体化，加快形成中心城市、大县城、小城镇、新农村“四位一体”发展格局。坚持“两手抓、两手都要硬”，深入开展文明城市、卫生城市、园林城市、食品安全城市“四城同创”。推动文化事业和文化产业大发展、大繁荣，促进物质文明和精神文明协调发展。坚持军民、军地深度融合，巩固全国双拥模范城成果，促进经济建设与国防建设协调发展。

(三)坚持绿色发展，大力改善生态环境。牢固树立“绿水青山就是金山银山”和“环境就是民生，青山就是美丽，蓝天也是幸福”的发展理念，坚持绿色富市、绿色惠民，大力推进生态文明建设，加快形成绿色发展方式和生活方式，实现生产空间集约高效、生活空间宜居适度、生态空间山清水秀。加强生态绿化保障建设，推动低碳循环发展，加强节能减排，促进资源节约高效利用，加强大气、水、土壤等环境保护和综合治理力度，全面完成“大气十条”和“水十条”的目标任务。加快建设资源节约型、环境友好型社会，促进人与自然和谐发展、永续发展。

(四)坚持开放发展，培育合作共赢新优势。深入实施开放带动战略，强化开放意识，提高开放素质，提升开放能力，以扩大开放带动创新、促进合作、推动发展，努力把运城建设成为新亚欧大陆桥、丝绸之路经济带上的重要节点，主动对接京津冀协同发展国家战略，深入推进晋陕豫黄河金三角区域合作，不断扩大合作成果。打造对外开放平台，充分发挥开发区承接产业转移的窗口作用。加快航空口岸建设，进一步发挥海关和检验检疫的作用，提升开放口岸功能。坚持把招商引资作为扩大开放的重中之重，坚持不懈开展集群化、专业化、产业链招商，提高招商引资实效，努力在寻求合作中扩大对外开放，在扩大开放中拓宽发展空间。

(五)坚持共享发展，努力保障和改善民生。坚持以人民为中心，持续增进民生福祉，实现社会公平正义，让全市人民在共建共享发展中有更多获得感。坚持从解决人民群众最关心最直接最现实的利益问题入手，加强教育、医疗、就业和社会保障等公共服务，不断提高公共服务共建能力和共享水平。坚持居民收入增长和经济增长同步，劳动报酬增长和劳动生产率提高同步，提高城乡居民收入水平。坚持把脱贫攻坚作为第一民生工程，实施精准扶贫、精准脱贫，坚决打赢脱贫攻坚战，确保全市22.7万贫困人口如期脱贫。

(六)坚持廉洁和安全发展，着力营造良好发展环境。牢固树立“发展必须廉洁，廉洁促进发展”的理念，严格落实“两个责任”，深入推进“六权治本”，把权力关进制度的笼子，培育廉洁发展的社会氛围。强化安全红线意识，始终把安全生产作为生命线、高压线、责任线，按照“党政同责、一岗双责、失职追责”要求，健全公共安全保障体制，严格落实安全监管责任，抓好安全隐患排查治理，加大安全考核问责力度，促进安全生产形势持续稳定好转。加强社会治安综合治理，完善社会治安防控体系，切实维护人民生命财产安全，保持社会和谐稳定。

各位代表，强化创新、协调、绿色、开放、共享、廉洁和安全发展理念，如期全面建成小康社会，承载着全市人民对幸福生活的美好向往和热切期盼。《纲要 (草案)》经这次人代会审议通过后，我们将全力抓好落实。我们坚信，经过全市人民的共同努力，全面建成小康社会决胜阶段的各项目标任务一定能够如期实现!三、2016年工作安排

2016年是“十三五”开局之年，也是推进结构性改革的攻坚之年。我们要全面贯彻党的十八届五中全会精神，认真落实中央、省经济工作会议、省委十届七次全会精神，按照市委三届七次全会和市委经济工作会议的部署，积极认识、适应、引领经济发展新常态，坚持稳中求进工作总基调，坚持稳增长、调结构、惠民生、防风险，坚持不懈狠抓“三个一百”，坚定不移落实“三个方案”，着力实施“三动三新”战略，着力抓好供给侧结构性改革，去产能、去库存、去杠杆、降成本、补短板，着力促进“六大发展”，加快建设新型工业大市、现代农业强市、文化旅游名市和晋陕豫黄河金三角区域性中心城市，努力实现“十三五”时期经济社会发展的良好开局。

综合考虑各方面因素，2016年我市经济社会发展的主要预期指标是：地区生产总值增长6.5%左右，固定资产投资增长12%，社会消费品零售总额增长 5.5%左右，一般公共预算收入增长3%，城镇居民人均可支配收入增长7%，农村居民人均可支配收入增长7%以上，居民消费价格涨幅控制在3%左右，城镇新增就业岗位4.2万个，城镇登记失业率控制在4.2%以内。约束性指标，按省下达我市的目标任务，确保全面完成。

重点抓好以下几方面工作：

(一)以“三个一百”为主抓手，不断强化项目支撑。一是加大“三个一百”推进力度。落实国家、省各项减负政策，千方百计帮助企业“降成本”;强化领导包联责任，保证项目建设顺利推进;加大协调推进力度，确保招商引资签约项目尽快落地。二是抓好重点项目前期工作。今年要全面启动“十三五”规划确定的基础设施、民生公共服务、生态环境建设等重大项目的前期工作。要扎实做好“冬季行动”实施方案确定的南同蒲铁路侯马至风陵渡段电气化改造、华泽铝电2×35万千瓦低热值煤发电、高铁商务区金融商务总部等91项重点项目的前期工作，实行“一个项目、一位领导、一套班子、一个方案、一抓到底、直至投产”的工作机制，完善和强化项目台账管理，缩短重点项目的立项、环评、规划等前期手续办理时限，为项目顺利开工创造条件。三是加快重点领域投资和重大项目建设。按照全省十大重点领域投向，注重生产类项目特别是工业项目和产业链项目的投资建设，结合我市的产业基础，着力推进装备制造、新能源、新材料、食品医药、文化旅游和现代服务业等领域投资。加快推进城市道路交通、棚户区改造等一批市政公共设施和民生项目建设。四是加大项目和政策争取力度。加强与国家部委、省直有关部门的沟通对接，争取运城-三门峡客专、运城-济源铁路、运城-阳城高速公路、风陵渡电厂、河津电厂、运城煤电铝材一体化、运城中西部地区装备制造业基地、运城“智慧城市”等重大项目获得支持。

(二)瞄准主攻产业板块，加快工业集群化步伐。一是坚定不移推进产业集群发展。依托工业园区和集群龙头企业，围绕产业链横向配套和纵向延伸，积极承接产业转移，毫不动摇落实“10、30、10”年度目标任务，深入推进园区化发展集群化招商。根据国家产业政策和县域产业基础、发展现状，适时对主攻产业板块进行动态调整、进退流动、差异化考核。在全面推进产业集群发展的同时，重点抓好汽车运输装备、医药、铝工业和轨道交通等产业集群。今年这四个集群在引进龙头企业上要有实实在在的突破。汽车运输装备产业以大运为龙头，积极与国内外整车企业合作，调整产品结构，大力发展新能源汽车。空港和运城开发区要积极帮助大运拓展市场，鼓励支持发展特种车，将新能源汽车优惠政策落实到位。着力引进汽车整车和汽车零部件企业入驻园区，由此带动汽车和运输装备制造业发展。医药产业以亚宝、石药银湖为龙头，进一步延伸产业链，加大招商引资力度。与国新能源在运城发展中药材基地相结合，发展种植、加工、交易、医药制造等上下游产业相互衔接、配套的产业集群，把医药产业集群做大做强。围绕产业链、集群化招商，积极与国内外行业中介组织合作，大力发展高端产品，做大体量。铝加工产业以华泽、华圣等为龙头，发挥煤电铝材产业优势，抓住国务院确定我省为全国首批电力体制改革综合试点省和国家工信部批准我市作为全国铝工业示范基地的重大机遇，加强与中铝、同煤等大集团的联合，吸引集聚一批实力强、产品科技含量高的国内外铝深加工企业，发展壮大铝深加工产品;支持重点铝加工企业加强与科研院所的合作，针对高端铝产品加工的技术瓶颈，进行攻关，努力在铝深加工前沿技术上有新的突破;争取大用户直供电试点，探索建立局域电网，推动电铝联营，降低用电成本，坚定不移推进煤电铝材一体化发展。轨道交通产业以中车永济电机为龙头，紧紧围绕铁路牵引电传动装置、轨道交通电传动系统和风力发电系统产业化基地建设，积极培育配套加工企业，打造山西南部的轨道交通产业基地。同时，大力支持和积极推进新材料、新能源等产业集群。二是下大力气改造提升传统产业。钢铁行业鼓励企业加大研发投入，增强新产品开发能力，加快新技术、新工艺、新装备应用，积极调整产品结构，重点发展工业加工用钢和无缝钢管等高端钢材品种，全面推进产品、质量和效益提升。继续加强与省内外有实力的大型钢铁集团开展交流与合作，全面提升钢铁行业核心竞争力。加快推进海鑫钢铁的复产及升级改造，争取早日恢复200万吨建筑用钢材生产，年底启动板材生产。水泥行业引导威顿、冀东等水泥企业利用先进适用技术，不断节能降耗，降低生产成本，增强竞争优势，扩大营销力度，提高产能利用率。焦化行业严格控制产能扩张，继续推动企业兼并重组。加快焦化行业装备和技术水平的优化升级，把更多精力投入到新产业的培育上。同时，坚决依法依规淘汰关闭落后产能，积极稳妥处置“僵尸”企业，推进其重组整合或退出市场。三是深入推进“两化”融合。围绕九大产业集群重点推进“两化”融合工程，培育2家国家级“两化”融合贯标示范企业、3家—5家省级示范企业，打造行业“两化”融合示范标杆。加大政策倾斜力度，扩大信息化建设范围，补齐信息化建设短板;引进中兴、金蝶等信息化龙头企业，引领我市企业信息化建设，提高全市“两化”融合水平。狠抓工业投资，今年实施工业项目255项，总投资1797亿元，年内完成投资379亿元。

(三)坚持“四化”引领，提高农业现代化水平。一是推进土地规模化。在全面完成土地确权颁证登记工作的基础上，深化农村产权制度改革，依法有序引导土地流转，发展多种形式的农业适度规模经营，不断优化土地资源配置，提高劳动生产率。二是加快组织企业化。新发展农民专业合作社500个，新培育示范社 180个，新发展家庭农场200家。扶持永济粟海、稷山晋龙、芮城温氏、闻喜牧原等重点企业发展，辐射带动一批种养殖小区和规模种养殖大户。三是提升技术现代化。稳定粮食生产。实施果品提质增效工程，加快现代果业标准园区建设，新增10万亩出口水果基地。加大新品种、新技术的示范、推广、应用力度，不断提高科技贡献率，着力解决农业技术化程度不高的短板问题，推动农业提质增效。再培训新型职业农民12000人，使村村有产业致富的“带头人”，户户有技术应用的“明白人”。四是扩大经营市场化。以企业为主体，大力推进农产品标准化、优质化、特色化、产业化、规模化和品牌化进程，尽快打造一批具有市场影响力的农业品牌。发展壮大农村经纪人队伍。做好农超对接、农市对接、农产品进社区以及农产品电子商务工作。新建10个惠农服务中心，为农民提供代耕、代种、代收、代储等保姆式、菜单式服务。在继续组织企业参加全国各地的农交会、展销会基础上，办好我市首届国际果品交易博览会。借鉴去年上海水果推介会的成功经验，组建整合各类营销队伍到全国各地推介运城的特色农产品，在北京、上海、广州、深圳等大中城市建立10个果品直销窗口。特别要以苹果出口美国为契机，打造优势品牌，扩大水果出口量，增加果农收入。五是加强农业基础设施建设。继续加强农田水利建设。全年新增和改善灌溉面积23.4万亩。北赵引黄二期工程实现主体完工、部分通水。小浪底引水和西范灌区东扩工程完成年度建设任务。完成土地治理10万亩。扎实推进农村人居环境改善“四大工程”建设，重点抓好“气化运城”100个新农村试点建设，创建25个省级、26个市级、53个县级美丽宜居示范村和1150个乡村清洁达标村。同时，摸清家底，搞好第三次全国农业普查工作。六是加快脱贫攻坚。坚持把脱贫攻坚作为最重大的民生工程，紧紧围绕“到2020年全市5个贫困县全部摘帽，贫困人口全部脱贫”的总目标，按照 “六个精准”的要求，全力抓好产业开发、易地搬迁、教育助学、生态补偿、社保兜底“五个一批”。全年实施易地扶贫搬迁8696人，减贫6万人。要咬定目标不放松，坚决打好脱贫攻坚战，决不落下一个贫困村、一个贫困户!

(四)发挥中心城市龙头作用，稳步推进新型城镇化。按照全省打造晋南城镇群的要求，围绕建设黄河金三角区域性中心城市的目标，坚持“四化”互动、“六化”衔接和中心城市、大县城、小城镇、新农村“四位一体、功能各异”，加强与首创集团、中建交集团、省建工集团、泛华集团等有实力、有影响的大集团的战略合作，创新城市建设投融资模式，优化新型城镇化空间布局。“十三五”期间，中心城市将采取PPP模式实施道路桥梁、地下综合管廊、城市水系、公共服务场馆、供热和城中村改造“六大系列工程”，今年启动实施42个重点项目，总投资118.8亿元，力争到“十三五”末使中心城市的功能更加完备、特色更加鲜明、风貌更加独特、市民生活更加便捷。

城市功能完善方面，通过建设河东西街、中银北路、舜帝街立交桥等道路桥梁和地下综合管廊，不断完善“九纵九横”城市骨干路网结构。新开工和启动道路项目15个，投资53.2亿元，新开工和启动地下综合管廊19个，投资30.7亿元。启动樊村、八一、安邑三大水库的综合治理和北郊干河改造，形成“一池四湖五渠”的城市水系格局，打造城市滨水绿色廊道和生态保护屏障。今年开工和启动水系项目5个，投资21.9亿元。完善城市集中供热体系，不断提高集中供热普及率，启动关铝热电厂可新增供热能力300万平方米以上的1号机组高背压改造。产业空间拓展和土地集约利用方面，完善城市设计，加大“城中村”和棚户区改造，发展高端产业，提升产业层次，提高单位面积投资强度，使有限的土地容纳更多的产业。加快东部新区、高铁片区建设，形成示范效应。积极化解商品房库存，盘活闲置房。扩大住房有效需求，落实住房信贷政策，发挥住房公积金作用。加快户籍制度改革，鼓励外地人和农村人口在城市购房落户。市民方便宜居方面，分步启动实施大剧院、科技馆、图书馆、规划馆、美术馆和市民服务中心等公共服务场馆设施建设，抓好小游园、停车场、农贸市场、公共厕所的规划和建设，方便市民生活。今年中心城市开工和启动公共服务设施项目群3个，投资13亿元。强化城市管理方面，深入开展文明城市、卫生城市、园林城市、食品安全城市“四城同创”。严厉打击违规违法建设行为。集中开展环境卫生、交通秩序、占道经营、夜市摊点等方面的专项整治。发挥城市社区作用，推行网格化服务管理。智慧运城建设方面，加快智慧运城“一平台五应用”建设力度。推进互联网、电信网、广电网“三网融合”，运用大数据、云计算、物联网、移动互联网等新一代信息技术，整合公共服务平台，实现各职能部门数据共享和城市的精细化、智能化管理。大力推动“互联网+”制造、农业、金融、民生服务、商贸物流、交通、文化旅游等发展。探索建设智慧城市产业园，推进以信息产业为代表的智慧产业集群化发展，引领新产业、创造新产品、满足新需求。

(五)培育壮大文化旅游产业，带动第三产业快速发展。围绕建设全省的文化旅游名市，加大文化旅游创意策划、宣传促销和市场化开发力度，狠抓龙头景区建设，加快文化旅游产业快速发展。一是做大“古中国”文化旅游品牌。深入挖掘尧舜禹文化中蕴涵的社会主义核心价值观文化基因，进一步增强全社会对“古中国” 的认同感，叫响叫亮“古中国”旅游目的地品牌，牵头做好山西“古中国”文化节系列活动。搞好文化旅游创意策划，引进高层次、专业化、有实力的投资商进行顶层设计，将我市丰富的文化旅游资源转化为可供人们体验、观赏、消费的文化旅游产品。突出关公文化和盐文化主题，全力推进河东盐池自然景观与关帝庙人文景观整合的龙头景区建设，连片规划、同步开发、一体推进、景城融合，努力打造盐湖国家级生态文化旅游度假景区、全球华人祭拜朝圣地、“古中国”国际旅游目的地。大力推进关圣文化建筑群申遗，连片打造关帝庙-常平家庙-关公圣像国家5A级景区。各县(市、区)也要明确打造1个龙头景区。二是强化旅游宣传推介。优化设计旅游线路，加大在黄河金三角、通航城市、通高铁城市、长三角、珠三角、沿海城市等主要客源地的宣传推介力度，做到高铁线路、重点城市、知名景区 “三个全覆盖”。三是开发旅游新业态。发挥运城农业资源丰富、自然风光秀美的优势，大力发展乡村旅游、休闲旅游、度假旅游、养生旅游、养老旅游、健康旅游、研学旅游、体育旅游、购物旅游、房车旅游等新型旅游业态。推行微旅新模式，推动互联网与文化旅游产业深度融合。加强在微博、微信、门户网站等新媒体平台上的旅游宣传，主动与国内知名旅游网站洽谈对接。四是强化区域旅游合作。加快启动“丝绸之路·黄河金三角旅游联盟”，积极对接和融入“丝绸之路”文化旅游重点地区，继续推行三省四市“一证游”惠民政策。创新举办“晋陕豫黄河金三角网络旅游节”，合力打造黄河风情游、根祖文化游、特色山水游、宗教文化游、休闲养生游等精品旅游线路。五是加快文化旅游市场化运作。稳步推进旅游行业体制机制改革，积极推进景区管理权、经营权分离的管理新模式。引进有实力、专业化的大型高端旅游公司参与文化旅游资源开发，尽快提升产业层次，不断激发发展活力。六是加快发展商贸物流业和现代服务业。推进32个商贸物流业项目建设。加快商业综合体建设，今年确保2个投入运营，5个主体封顶、5个开工建设。建设100个放心早餐网点、培育5家中小家政服务企业、增加20个平价蔬菜店、改扩建3个农贸市场。抓好大型农产品物流项目。重点建设润恒现代农副产品、晋善晋美和晋南农产品商贸物流园等项目，建设10个具有分级配送功能的直营乡镇商贸中心。引导和支持大中型流通企业、连锁公司将经营触角向农村地区延伸，改善农村市场购物环境，进一步扩大农村消费。大力发展房地产服务业、健康服务业、养老服务业等现代服务业和电子商务、金融保险、文化创意等新兴产业。以星河创业创新基地为示范，推进大众创业、万众创新，力争在全市建成两个省级众创空间。

(六)坚持绿色发展，加快生态文明建设。一是抓好环保问题的整改。对环保部华北环境督查中心挂牌督办的34个问题，要采取过硬措施，一一对照，逐个整改，确保今年4月底前全面整改到位，6月底接受国家环保部验收。二是持续开展造林绿化。全力以赴抓好造林绿化各项重点工程落实，确保全省林业现场会在我市圆满召开。全年完成造林20万亩以上。搞好高速公路的通道绿化提档升级。全力抓好经济林产业富民提质增效，努力创建国家级森林城市和湿地自然保护区。高标准完成350个园林村庄建设。继续加快全市园林绿化建设步伐，新增绿化面积100万平方米，其中，中心城市增加20万平方米。中心城市和永济市申报国家级园林城市，河津市、芮城县启动省级园林县城创建工作。三是抓好节能减排和大气、水污染防治。围绕节能目标和节能产业两大主攻方向，强化节能考核，严格奖惩兑现。积极培育节能产业，建设全省高效节能电机生产基地，发展节能产品、新能源汽车、光伏发电等相关产业。在工业、建筑、交通、公共机构和商贸等领域推广先进适用节能技术，提高节能降耗水平。强力推进焦化、电力、供热等重点行业、重点企业的达标改造，确保污染物稳定达标排放。抓好建筑施工扬尘、道路扬尘污染控制，加强黄标车、老旧车淘汰及限行，抓好燃煤锅炉整治。环保、水务、林业、住建等部门要密切配合，综合施策，加大汾河、涑水河流域环境综合整治力度，地表水环境质量达到省定考核要求。继续加强饮用水水源地保护，确保城市集中式饮用水源地取水水质达标率达到100%。积极推广生物技术，科学减量使用化肥、农药等农业投入品，发展循环农业，减少面源污染。生态环境没有替代品，用之不觉，失之难存。我们一定要坚定信念，坚定不移推进绿色发展，推动自然资本大量增值，让良好的生态环境成为人民生活的增长点，成为展现运城发展形象的发力点!

(七)实施“三动三新”战略，不断释放发展活力。一是深化各项改革。继续深化行政审批制度改革，尽快启动网上行政审批，建成实体性市民服务中心，简化审批手续，规范审批行为，优化审批流程，提高行政效能。全面推行权力清单、责任清单和负面清单制度，并逐步向乡村延伸。转变政府职能，理顺权责关系，优化组织结构。分类推进事业单位改革，全面启动城市公立医院改革。积极推进国企改革。深入推进商事登记制度改革，释放民营经济活力。深化财政制度改革，扩大预算公开范围，规范地方政府债务管理。二是扩大对外开放。构建对外开放大格局，加强对外开放的顶层设计，进一步理顺完善推动开放的工作机制，全面提高各级干部开放的意识、素质和能力。深化晋陕豫黄河金三角区域合作，不断健全省级协调机制、“四市”联席会议等合作体制机制。抢抓国家加强生态环境建设战略部署，以黄土高原生态建设和黄河中游生态综合治理为突破口，尽快启动一批实质性的合作项目。推进跨区域互通公交、医疗结算、师资共享等公共服务平台建设，让群众切切实实感受到区域合作的实惠。全力做好外贸进出口工作，提高经济外向度。三是加大招商引资力度。在力促100个重点招商引资项目签约落地的前提下，围绕产业集群、现代农业板块和文化旅游产业等重点，瞄准珠三角、长三角、环渤海等重点区域，开展点对点、小分队、专业化、集群化、精准化招商，使全市在招商引资项目签约率、履约率和落地率上有较大突破。加快航空口岸开放步伐，力争把空港经济开发区建成承接产业转移和新型业态的重要平台，建成黄河金三角全新开放的物流和交通枢纽。适时申请设立综合保税区。四是强力推进“三个突破”。科技创新方面：加大科研投入，逐步提高科技支出占财政支出的比例。积极引进高层次的管理人才和科研人才，设立企业研发中心。探索建设省级高新技术产业开发区，培育一批产业前景良好的科技型中小企业，打造引领经济发展新常态的创新型产业集群。加快山西运城国家农业科技园区建设步伐。以建设国家级高技能人才培训基地为契机，注重与工业园区、产业集群用工需求相衔接，针对性地搞好职业技能培训，培养与我市产业集群发展相适应的各类实用技术人才。深化产学研合作，继续加强与中科院北京分院、西北工业技术研究院、西北工业大学等科研院所的合作，提高科技成果转化率。全年新增高新技术企业10家，累计突破70家。积极筹建科技大市场，整合各类科技资源，促进科技成果转变，加快创新驱动步伐。深入推进“品牌兴市”战略，支持引导各类企业争创驰名、著名商标。民营经济发展方面：全面落实国家、省支持民营经济发展的各项政策措施，开展民营企业项目大起底工作，在市场准入、信贷支持、上市融资、人才引进、参与国有企业改革以及项目落地开工建设等多方面给予政策倾斜。大力实施中小微企业成长工程和创业基地建设。今年培育认定省级中小企业创业基地1家、市级2家;建成与国家和省对接的市级中小企业公共服务平台，为广大中小微(民营)企业提供全方位公共服务。加快企业股份制改造，对已经筛选出的100家企业，分期分批全面进行股份制改造，帮助企业完善制度、规范管理，为进入资本市场奠定基础。推进诚信体系建设，开展企业管理大提升行动，引深中小企业规范化管理提升行动，推动民营经济振兴行动。加强企业家队伍建设，制定培训计划，邀请高水平、高层次的专家教授为我市的企业家授课，开阔眼界，提升企业家的思维能力和应对市场风险的能力。金融振兴方面：大力推进企业挂牌和上市工作，完善政策性融资担保机构，并将这两项工作纳入年度考核目标任务。建立政银企长效合作机制，争取省内外金融机构更多的信贷支持。整合各方资源，筹划建设企业项目资金对接平台。坚持“一企一策”解决企业债务问题，防范和化解金融风险。银企双方要相互理解，增强互信，抱团取暖，共渡难关。加快农信社改制和“险资入运”步伐。继续发挥好“企业应急转贷资金平台”的作用。用足用活用好产业引导基金，引导和撬动更多社会资本支持我市工业集群化、农业现代化、新型城镇化、信息化、文化旅游产业和稳增长调结构“三个一百”发展。依法严厉打击逃废债务行为，规范金融市场秩序，创优金融环境，努力建设现代金融企业集聚区，坚定不移打造黄河金三角区域金融中心城市。

(八)全面发展社会事业，着力保障和改善民生。千方百计促进就业创业。坚持实施就业优先战略，实施更加积极的就业政策，落实创业扶持政策，用好创业创新引导资金，全力推动大众创业、万众创新，鼓励发展众创、众包、众扶、众筹空间，促进以创业带动就业。加强就业培训，推行终身职业技能培训制度，实施职业培训全覆盖计划，对重点人群和困难人群免费进行职业培训。全面发展教育事业。针对中心城区中小学幼儿园建设历史欠账问题，分期分批下大气力补齐短板，跟进落实大运学校、国际学校、理想学校、盐湖区实验小学北校区、五洲观澜双语幼儿园、金海湾幼儿园等校园建设，实实在在解决好中心城区入学难、入园难问题。新改扩建农村公立幼儿园28所，高标准完成河津、新绛、闻喜、盐湖等4个国家义务教育均衡县达标验收。建立中小学教师补充长效机制，逐步解决教师编制问题。着力促进基础教育均衡发展，努力提高教育质量。进一步抓好七大职业教育集团建设，为推动经济社会发展，提供雄厚的人才支撑。大力发展医疗卫生事业。深化医疗卫生体制改革，全面推进城市公立医院改革，实现医疗、医保、医药联动，扩大省内外异地就医直接结算范围，更好地满足城乡群众基本医疗服务需求。加强基层医疗设施和人才队伍建设，提升医疗服务水平。大力推进中医药发展。运城市第一医院年内全面完成土建工程，启动实施市儿童医院和中心医院二期项目建设。完善分级诊疗实施方案，稳妥推进分级诊疗工作。全面落实一对夫妇可生育两个孩子政策。推进文化体育事业。加快完善公共文化服务体系，开展丰富多彩的群众文化活动。深入推进文化惠民工程和戏曲惠民活动。加强非遗名录体系建设和非遗传承人培训工作。开展全民阅读活动，引深“书香运城”建设。大力开展群众性体育活动，加快体育设施建设，提高体育设施开放率。加快体育产业发展，促进体育消费增长。全面加强和改进档案工作，积极推进市档案馆和县级馆项目建设，稳步推进数字化档案馆室体系建设。强化社会保障。积极落实全民参保计划，扎实推进机关事业单位、企业基本养老保险制度并轨运行，继续提高退休人员基本养老金待遇水平。完善城乡最低生活保障制度，畅通社会救助申请受理渠道，鼓励社会力量参与社会救助。全面开展重特大疾病医疗救助。加大特困人员供养服务机构建设力度，提升集中供养能力和质量。新建130个农村老年人日间照料中心;开展城市老年人日间照料中心试点工作。建立适度普惠型儿童福利制度。新增公共租赁住房120套，城市棚户区改造1.2万套，基本建成公共租赁住房和棚户区改造7000套;完成农村危房改造9220户。完成省下达我市解决农村人口饮水安全任务。

(九)坚持公平正义，全力维护社会和谐稳定。加强民主法治建设。自觉接受人大及其常委会监督，认真执行人大各项决议。积极支持人民政协履行政治协商、民主监督和参政议政职能。坚持重大事项向人大报告、向政协通报和重大决策提交人大审议制度。充分听取各民主党派、工商联、人民团体和社会各界意见，认真办理人大代表建议和政协委员提案。积极支持人民法院、检察院依法履行职责。建设完备的公共法律服务体系，普遍建立县乡公共法律服务中心，扩大法律援助覆盖面，全面建立法律顾问制度。全面启动“七五”普法，强化全民法治观念，加快依法治市进程。加强精神文明建设，不断提升市民文明素质和社会文明程度。加强和创新社会治理。建立完善社会调处化解矛盾纠纷综合机制。加强信访工作，创新体制机制，健全完善畅通有序合法的诉求表达渠道，积极解决人民群众的合理诉求。加强社会治安综合治理，着力构建立体化社会治安防控体系，有效防范和妥善处置各类群体性事件，深入推进打黑除恶专项斗争，全力维护社会和谐稳定。高度重视安全生产。全面推进安全生产党政同责“五级五覆盖”。实行严格的安全生产目标考核，加强责任追究。严格落实安全生产“两个主体”责任，切实抓好煤矿、非煤矿山、尾矿库、消防、防汛、道路交通、森林防火、危险化学品、烟花爆竹等重点领域、特种设备以及学校、商场等人员密集场所的安全隐患排查治理，确保安全生产形势持续稳定好转。积极实施食品安全战略，强化食品安全责任制，继续加大创建食品安全城市工作力度，严厉打击食品、药品违法犯罪行为。整治非法超限超载运输行为。

同时，加强应急管理和防灾减灾能力建设，切实提高城乡基础设施和房屋建筑抗震能力。统筹经济建设与国防建设、富民与兴武协调发展。增强全民国防观念，加强国防后备力量建设，做好优抚安置和新一轮全国双拥模范城创建工作，以实际行动积极支持国防和军队改革，推动军民融合深度发展。支持工、青、妇工作，充分发挥群团组织的作用，发展儿童、老龄、残疾人和红十字会等事业，做好民族宗教、对台、气象、科普、史志等工作。

(十)加强政府自身建设，全面提高政府效率。坚持“32字”工作导向，全面加强政府自身建设，“马上就办，真抓实干”，推进政府效率大提升。一是加强党风廉政建设，推进政务公开。认真履行全面从严治党主体责任，从严落实党风廉政建设“一岗双责”，严格执行中国共产党党章、廉洁自律准则和纪律处分条例等各项规定。全面落实“六权治本”，使之成为规范政府行为的铁律。严格控制“三公”经费，厉行节约，反对浪费。强化行政监察和审计监督，围绕国土、交通、房地产等重点领域和资源配置、工程招投标、政府采购、资金分配等关键环节，严肃查处各类违纪违法行为，坚决遏制腐败现象滋生蔓延。全面推进行政决策、执行、管理、服务和结果公开，努力建设法治政府、创新政府、廉洁政府和服务型政府。二是加强学习培训，提高素养能力。干部的素养和能力，直接关系发展的水平和效果。各级政府干部要针对能力危机和本领恐慌问题，自觉加强学习，提高专业思维、专业素养和专业能力，加强对经济、政治、文化、社会、生态等方面知识的学习，加强理论、政策、科技、管理、法律法规的学习，加强对新兴产业、先进制造业、现代服务业、新型工业化、新型城镇化、农业现代化等方面知识的学习，加强对充满时代气息的新技术、新业态的了解，努力提高驾驭现代经济发展的本领，成为经济社会管理的行家里手。三是加强作风建设，解决为官不为。坚持问题导向，直面基层一线，拿出更多的时间和精力深入调查研究，现场解决问题。针对“为官不为”问题，采取有效措施着力解决能力不足“不能为”、动力不足“不想为”、担当不足“不敢为”的现象。利用“政府大讲堂”、行政学院等平台，加强对干部的精准教育培训，突出针对性和实效性，消除干部的知识空白、经验盲区、能力弱项，以解决能力不足问题。在严格管理的同时，通过政治上激励、工作上支持、待遇上保证、心理上关怀，以解决动力不足问题。要把干部在推进改革发展中因勇于探索出现的错误失误同违纪违法、谋取私利的行为区分开来，为敢担当的干部担当、为敢负责的干部负责，以解决不敢担当的问题，充分调动干部干事创业的积极性。四是加强督查问责，狠抓工作落实。坚持重点督查、随机督查和定期督查相结合，对重点工作、重点项目的推进落实情况，一项一项督办，实行一月一汇总，两月一督查，一季一推进，年终总考评。加大统计监测力度，对符合条件的投资项目，切实做到及时入库，防止漏统，更要防止虚报。进一步创新政府运转机制，提高工作效率。强化落实意识，抓好绩效考核，严格奖惩兑现。凡定下的事，就要紧盯不落实的事，问责不落实的人，严查不作为的官，确保各项工作落地生根，取得实效。

各位代表，2016年的目标任务已经明确，形势逼人、任务艰巨、使命光荣。让我们紧密团结在以习近平同志为总书记的党中央周围，在中共运城市委的坚强领导下，团结一心，开拓进取，扎实工作，为实现“十三五”良好开局，为建设新型工业大市、现代农业强市、文化旅游名市和晋陕豫黄河金三角区域性中心城市作出新的更大的贡献!
